# Molecular Mechanisms of Noncoding RNA in the Occurrence of Castration-Resistant Prostate Cancer

**DOI:** 10.3390/ijms24021305

**Published:** 2023-01-09

**Authors:** Yu Lin, Haisong Tan, Guopeng Yu, Ming Zhan, Bin Xu

**Affiliations:** 1 Department of Urology, Shanghai Ninth People’s Hospital, Shanghai Jiao Tong University School of Medicine, Shanghai 200011, China; 2 The Core Laboratory in Medical Center of Clinical Research, Department of Molecular Diagnostics & Endocrinology, Shanghai Ninth People’s Hospital, State Key Laboratory of Medical Genomics, Shanghai Jiao Tong University School of Medicine, Shanghai 200011, China

**Keywords:** castration-resistant prostate cancer, long non-coding RNAs, micro RNAs, androgen receptor, tumor metabolisms, epigenetic modifications

## Abstract

Although several therapeutic options have been shown to improve survival of most patients with prostate cancer, progression to castration-refractory state continues to present challenges in clinics and scientific research. As a highly heterogeneous disease entity, the mechanisms of castration-resistant prostate cancer (CRPC) are complicated and arise from multiple factors. Among them, noncoding RNAs (ncRNAs), the untranslated part of the human transcriptome, are closely related to almost all biological regulation, including tumor metabolisms, epigenetic modifications and immune escape, which has encouraged scientists to investigate their role in CRPC. In clinical practice, ncRNAs, especially miRNAs and lncRNAs, may function as potential biomarkers for diagnosis and prognosis of CRPC. Therefore, understanding the molecular biology of CRPC will help boost a shift in the treatment of CRPC patients. In this review, we summarize the recent findings of miRNAs and lncRNAs, discuss their potential functional mechanisms and highlight their clinical application prospects in CRPC.

## 1. Introduction

Prostate cancer (PCa) is the most prevalent male-related malignancy in the western world and is driven largely by androgen receptor (AR) signaling [1,2]. Given this, androgen deprivation therapy (ADT), including chemical and surgical castration, is the first line of defense for men with advanced prostate cancer and an important adjunctive therapy to radiotherapy for patients with medium- and high-risk prostate cancer [3,4]. Despite responsiveness, however, relapse almost invariably occurs as the cancer evolves toward the lethal phenotype of the disease: so-called castration-resistant prostate cancer, for which there is no effective medication [5]. As a result, despite its slow course, PCa still ranks third in cancer-related mortality in the United States [1]. CRPC is a highly heterogeneous disease that involves several molecular mechanisms, including intratumoral androgen synthesis, the formation of AR splicing variants and the enhanced activity of AR coactivators [6,7,8,9,10]. In recent years, despite great advances in the treatment of CRPC, most patients with CRPC progress to lethal metastatic castration-resistant prostate cancer (mCRPC) and eventually show resistance to the FDA-approved medications abiraterone and enzalutamide, two classical anti-androgen drugs [11,12,13,14]. Therefore, a better understanding of the mechanisms that drive CRPC is crucial for clinical management. Recently, immune checkpoint inhibitors have been used successfully to treat several solid tumors, such as liver, cervical and breast cancers [15]. Additionally, immune checkpoint blockade (ICB) could be a novel therapeutic option for CRPC patients with bone metastases derived from the upregulation of IL-1ra in PCa [16,17]. For instance, a phase II clinical trial reported that nivolumab plus ipilimumab (anti-PD-1) has antitumor activity in patients with mCRPC [18].

For decades, studies on the molecular mechanism of prostate cancer have focused on protein-coding genes. Quite recently, the doctrine of molecular biology was changed with a deeper understanding of noncoding RNAs (ncRNAs). In recent decades, a series of studies have revealed the scientific secrets of ncRNAs, which have pivotal regulatory roles in almost all cellular and biological functions, such as apoptosis, migration, drug resistance [19], cellular metabolism [20], chromatin structure [21], RNA modifications [22,23] and translation regulation [24], making them a novel target for drug discovery. In addition, advances in ncRNA isolation and detection techniques have made them promising candidates for biomarker development. For instance, plasma exosomal miR-1290 and miR-375 were considered robust prognostic biomarkers for CRPC patients based on the significant correlation of these two miRNAs with survival [25]. Finally, ncRNAs can be therapeutically targeted through synthesis of complementary oligonucleotides to either enhance the stability or accelerate degradation of other molecules in clinical practice [26,27].

Historically, ncRNAs can be classified as short ncRNAs (snc RNAs, approximately 18–200 nt transcript) and long noncoding RNAs (lncRNAs, >200 nt transcript) based on their sizes. The most investigated ncRNA types include microRNAs (miRNAs), lncRNAs and circular RNAs (circRNAs) [28]. miRNAs are endogenous, small ncRNAs of approximately 18–22 nt in length that negatively regulate gene expression at the posttranscriptional level by base pairing with target mRNAs. In animals, miRNAs typically bind to the 3′ untranslated region (UTR) of their target mRNA through limited sequence complementarity [29], which leads to increased degradation of target mRNAs; however, stabilization of the target mRNAs has also been reported [30]. Consequently, molecular modes of miRNA-mediated translational repression mainly include the following: a single miRNA could target multiple mRNAs, and one mRNA can be targeted by several miRNAs cooperatively [31]. This classical action model makes miRNA mimics and miRNA inhibitors a class of promising therapeutic agents.

In contrast to miRNAs, lncRNAs are a group of transcripts with sizes greater than 200 nt that play regulatory roles at both the transcriptional and posttranscriptional levels [32,33]. Structurally, lncRNAs can be classified into five categories: sense lncRNAs, antisense lncRNAs, bidirectional lncRNAs, intergenic lncRNAs and intronic lncRNAs [34]. lncRNAs function through a variety of mechanisms which depend on their subcellular localization. A classic example is that cytoplasmic lncRNAs usually act as miRNA sponges to regulate the expression of mRNA targeted by miRNAs through competing endogenous RNAs (ceRNAs) [35]. In contrast, nuclear-localized lncRNAs could bind to specific transcription factors or chromatin-modifying complexes to regulate gene regulation indirectly [36].

In the present review, we attempt to clarify the major contribution of ncRNA-regulated mechanisms, including cancer metabolism, epigenetic modifications and abnormal AR signaling, to the occurrence of castration-resistant prostate cancer (Table 1).

## 2. CRPC and Its Mechanisms

The diagnostic criteria of CRPC status are serum testosterone <50 ng/dL or 1.7 nmol/L plus biochemical progression or radiologic progression [61]. Biochemical progression refers to three continuous increases in PSA 1 week apart, while radiologic progression refers to appearance of either new bone lesions or soft tissue lesions using the response evaluation criteria in solid tumors [62]. Despite serum testosterone deficiency, the occurrence and progression of CRPC are still largely dependent on androgens and the androgen receptor, usually due to the persistent interaction between androgen and AR [63]. The adaptive responses of the AR pathway in CRPC can occur as a result of excrescent androgens synthesis and androgen receptor changes, such as AR gene amplification, AR gene mutations and AR variants formation [64].

Androgens are not only involved in the normal development of the prostate gland, but also in the occurrence of PCa. Given this, androgen deprivation therapy (ADT), including chemical and surgical castration, is the first line of defense or an important adjunctive therapy to radiotherapy for men with PCa [65]. Despite responsiveness, however, relapse almost invariably occurs as the cancer evolves toward the lethal phenotype of the disease, so-called castration-resistant prostate cancer, for which there is no effective medication [5]. Androgen can be synthesized from adrenal androgens in prostate tissues by steroidogenic enzymes. ADT reduces serum DHT levels, but DTH can still be detected in CRPC [66]. Therefore, when ADT is performed, the remaining androgens synthesized from adrenal gland and tumor tissues are sufficient to activate androgen receptor [67]. Elevated AR expression is one of the most common changes in CRPC and related to the development of ADT resistance [68]. Increased AR levels also hypersensitize PCa cells to castration status, which can induce resistance to anti-AR drugs [69]. The elevated AR levels in CRPC are usually caused by AR gene amplification, with the amplification rate reaching about 50%, which depends on the different course of CRPC and therapy [68,70]. In addition, the amplificated AR gene could also be detected in CTCs (circulating tumor cells) of patients with CRPC [71]. Another contributing factor of CRPC is AR mutations, with a mutation rate of 5–30% [72]. However, studies have confirmed that AR mutation can be observed in primary PCa and increased after ADT treatment, which underlies the basis of ADT resistance [73]. Most common mutation sites of the AR gene are located in the ligation binding domain, which allows AR to be activated even at low androgens conditions [71]. In addition, mutations in the AR-NTD often cause changes that promote AR transactivation activity, such as altered recruitment of coactivators, increased response to androgens, enhanced AR stability and nuclear localization [71,74]. Additionally, studies have confirmed that AR variants (AR-Vs) are a driving factor of CRPC. Other studies have also suggested that current targeted drugs, such as enzalutamide, are unable to effectively inhibit AR activity driven by AR-Vs, which lacks AR-LBD (ligand-binding domain) [75,76]. It is well known that non-transcriptionally active AR-FL is located in cytoplasm where it interacts with Hsp90. However, some truncated AR-Vs, such AR-V7 and AR-V1, lack exon7, a Hsp90 binding site, and could be transported directly into the nucleus where they are constitutively active [77]. In addition to the ability of AR-Vs to locate in the nucleus independent of androgens, AR-Vs can also form dimers directly with AR-FL, leading to nuclear localization and thus binding to AREs [77,78,79].

## 3. Noncoding RNAs and Metabolic Remodeling in CRPC

Metabolic remodeling is considered a common hallmark of the biological capabilities of cancer obtained during the multistep initiation of human tumors, including PCa [80]. Currently, the most widely studied tumor metabolism is glucose metabolism abnormalities. A series of studies have revealed the close interactions between metabolic disorders and cancer biology, including tumor proliferation, metastasis and drug resistance [81,82,83]. Clinically, the treatment of mPCa (metastatic prostate cancer) and CRPC is a significant challenge because of the complex pathogenesis. Recent studies found that patients treated with ADT showed an elevation in bile acid levels [84], implying a correlation between ADT and lipid metabolism changes. Thus, there is a reason to suspect that lipid metabolism changes are a potential causative factor of castration resistance. In contrast, cancer biology could also influence metabolic processes. For instance, it is well known that the dysregulation of autophagy is closely related to cancer. Interestingly, studies have found that the significance of autophagy in cancer may be due to its potential effect on tumor metabolism regulation [85,86]. Thus, a better understanding of cancer metabolism is conducive to understanding the etiology and pathogenesis of tumors.

Given the key regulatory function of ncRNAs in cancers, ncRNAs have been reported to be involved in multiple cancers, including metabolic abnormalities [87]. A previous study found that the LncRNA CCAT2 regulates the expression of GAC (glutaminase isoform C), an alternative splicing isoform of GLS, uncovering the regulation of glutamine metabolism by lncRNAs. This modulation of cellular energy metabolism was achieved by interacting with the cleavage factor I (CFIm) complex in an allele-specific manner, which contributes to the malignant transformation and progression of CRC (colorectal cancer) [88].

Similarly, in prostate cancer, an androgen-induced prostate-specific LncRNA named prostate cancer gene expression marker 1 (PCGEM1) was discovered to increase glucose uptake and glycolysis for adequate energy supply and the proliferation and survival of LNCaP cells [89]. PCGEM1-mediated metabolism regulation was achieved via affecting several metabolic pathways, including glucose and glutamine metabolism, and functioning as a coactivator of c-Myc and a series of metabolic genes, making it a promising target for treatment intervention [89].

Previous studies have confirmed that AR signaling may limit glycolysis and enhance lipogenesis in PCa, hence driving the proliferation and migration of PCa cells [90,91,92]. Therefore, it is reasonable to assume that cellular metabolism alterations may contribute to the development and progression of CRPC due to the key function of AR in the tumorigenesis and progression of CRPC. However, currently, there is little research on the involvement of ncRNAs in metabolic abnormalities in CRPC. It is well-known that c-Myc may drive many metabolic changes, including simulating glycolysis, to meet the increased need for nucleic acids, proteins and lipids for an aggressive proliferative phenotype [93,94,95]. Recently, a report by Jay et al. confirmed that c-Myc is a direct target of miR-644a in PCa cells. Consistently, as a key enzyme in glycolysis, GAPDH expression was significantly suppressed by miR-644a by direct interaction with GAPDH which subsequently decreased its expression and enzymatic activity, thereby suppressing CRPC cells proliferation, indicating the inhibitory role of miR-644a in the Warburg effect [96]. Therefore, this result confirmed that miR-644a could influence the malignant behaviors of CRPC cells by regulating metabolism-related genes expression. Additionally, as a member of the Sp transcription factor family, it has become increasingly clear that Sp1 can lead to metabolic dysregulation and the progression of PCa [97,98]. Recently, a study indicated that high expression of Sp1 significantly increased glucose consumption and lactate production in CRPC cells [99]. Mechanistically, miR-361-5p can reverse regulate Sp1 by directly binding to the 3′-UTR of Sp1 mRNA, consequently inhibiting CRPC cell growth and glycometabolism in a Sp1-dependent manner [99]. Moreover, LncRNA MALAT1 plays a key role in PCa glycolysis and lactate through enhancing MYBL2 protein levels, thus driving PCa initiation and progression [100]. SREBP-1, a kind of sterol regulatory element-binding transcription factor involving in controlling lipogenesis and lipid metabolism, is currently reported to be transcriptionally regulated by miR-21 in PCa [101]. In addition, miR-21 inactivation also decreased the levels of FASN (fatty acid synthase) and ACC (acetyl-CoA carboxylase), and thus inhibited the proliferation and migration of human PCa cells [101]. GLUT1 (glucose transporter 1), a glucose and fructose transporter, mediates the basal-level uptake of glucose into various types of cells [102]. A recent study has showed that GLUT1 was a direct target of miR-378a. miR-378a hampered glucose metabolism and weakened proliferation in PCa cells via regulating mRNA levels of GLUT1 and may be a potential treatment target for highly aggressive glycolytic PCa [103]. Additionally, previous study has found that two miRNAs, miR-185 and miR342, could regulate lipogenesis and cholesterogenesis in PCa cells [104]. Mechanistically, miR-185 and miR342 could inhibit the expression of FASN (fatty acid synthase) and HMGCR (3-hydroxy-3-methylglutaryl CoA reductase), which are the target genes of SREBP-1, a critical regulatory factor for lipogenesis [104].

## 4. Noncoding RNAs and Epigenetic Dysregulation in CRPC

### 4.1. Noncoding RNAs and Epigenetics Modifications

In recent decades, biologists have become increasingly concerned about the epigenome. Epigenetic alteration has become a driving factor of malignant cell proliferation and the development of cancers. Conventional epigenetic aberrations at the transcriptional level include methylation and acetylation modifications of DNA and histones, as well as chromatin remodeling [105]. Moreover, with deeper research on ncRNAs and the development of novel sequencing techniques, scientists have found that epigenetic modifications exist in ncRNAs and regulate their expression level through various mechanisms [106,107]. A dozen years ago, researchers found that lncRNAs may be more competitive epigenetic regulation molecules than proteins, at least in the case of locus- and allele-specific control [108]. A series of experiments in recent years confirmed its epigenetic regulatory functions in RNA and proteins [109] through direct interaction with methylation-related enzymes or recruitment of chromatin modifying complexes, therefore influencing gene expression [110,111]. Additionally, ncRNAs are base modifications, which explains their abnormal expression [112,113,114,115,116].

### 4.2. The Interaction between ncRNAs and Epigenetics in CRPC

There is an increasing amount of evidence that regulatory ncRNAs play a significant role in epigenetic control, and these ncRNAs highlight the prominent role in gene expression. Recently, a novel LncRNA, LncRNA NXTAR, has been discovered to be repressed and been identified to act as a tumor suppressor in PCa [117]. LncRNA NXTAR could suppress AR expression via binding upstream of the AR promoter and recruiting EZH2, which marks the AR promoter with H3K27me3 repressive epigenetic marks [117]. This negative regulation on AR expression undoubtedly inhibited PCa cells proliferation and aborogated enzalutamide-resistant in PCa, therefore providing a therapeutic strategy for CRPC. PRMT5 is a protein arginine methyltransferase that catalyzes the majority of symmetric arginine dimethylation in many histone and non-histone proteins [118]. Recently, a study reported that the epigenetic regulator PRMT5 promoted PCa progression by inhibiting the transcription of CAMK2N1 and was mediated by miR-331-3p [119]. Serving as a ceRNA, circSPON2 could abrogate the repressive effect of miR-331-3p on its direct downstream target of PRMT5 and thus induce PCa cells proliferation and migration [119]. In addition to epigenetic modifications of genes and proteins that are affected by lncRNAs, epigenetic alterations are also involved in miRNA dysregulation associated with the development and progression of CRPC. It has been reported that miR-27a-5p was significantly upregulated in PCa cells in comparison to PNT2 [120]. Interestingly, LNCaP cells (an androgen-sensitive cell line) exhibited lower miR-27a-5p expression levels and higher promoter methylation levels, whereas in PC3 cells (an androgen-insensitive cell line), miR-27a-5p expression and promoter methylation levels had the opposite trends [120]. The low methylation levels of the miR-27a-5p promoter in PC3 cells allow c-Myc to bind to its promoter region and consequently positively regulate the expression of miR-27a-5p. Another study indicated the upregulation of miR-146a expression in both LNCaP and PC3 cells following 5-aza-CdR treatment, an inhibitor of DNA methyltransferase. Surprisingly, the miR-146a expression level in LNCap cells was significantly higher, whereas methylation levels were lower than those in PC3 cells, suggesting a delayed effect of miR-146a promoter hypomethylation on the progression of castration-resistant prostate cancer [121]. Several studies have already indicated that hypermethylation of the promoter is a major contributor to low expression of miR-124 in different tumors, including cervical cancer and pancreatic cancer [122,123]. Likewise, in another study, a DNA methylation analysis showed that the promoters of miR-124-2 and miR-124-3 were in a higher methylation status in AR-negative PCa cells than in AR-positive PCa cells [124]. However, the high methylation activity was only significant in DU145 cells, rather than in PC3 cells [124]. Unexpectedly, the expression of miR-124 has not changed considerably, which is inconsistent with the conventional view that DNA methylation leads to permanent gene silencing. One possibility would be that DNA methylation cannot be thought of as a “lock” for gene reactivation but provides a memory signal for long-term gene silencing [125]. Overall, these studies illustrated that the abnormal methylation status of certain miRNAs plays a vital role in the progression of androgen-dependent prostate cancer to castration-resistant prostate cancer.

### 4.3. Noncoding RNAs Related Signaling Pathway in CRPC

Cell signaling plays an important role in a variety of biological processes, including carcinogenesis. Numerous signaling pathways have been identified in CRPC, such as the WNT pathway [126,127], the PI3K/AKT/mTOR pathway and the Hippo/YAP pathway [128,129]. Recently, the prominent role of ncRNAs as essential signal transduction mediators in cancer signaling pathways has also emerged. The interactions between lncRNAs and key signaling mediators act as the major mechanism in cancer signaling pathway regulation [130].

PI3K/AKT signaling is among the pivotal pathways responsible for driving the process of malignancy in many solid tumors and is emerging as a potential target for cancer [131]. Shang et al. reported that lncRNA PCAT1 expression showed a significant increase in CRPC tissues compared with HSPC tissues via bioinformatics analysis, suggesting the positive relevance of lncRNA PACT1 to CRPC development or progression [132]. Carcinogenesis is accomplished by the activation of AKT and NF-kB signaling pathways in CRPC through increasing the expression of phosphorylated AKT and phosphorylated NF-kB p65 [133,134]. Mechanistically, lncRNA PCAT1 competitively inhibited the binding of PHLPP (PH domain leucine-rich repeat protein phosphatase) to FKBP51 (FK506-binding protein 51) by directly binding to FKBP51, a scaffold protein regulating the function of PHLPP and CHUK (conserved helix-loop-helix ubiquitous kinase), and therefore mediated the activation of AKT and NF-kB signaling [135]. Similarly, Daniela et al. reported that miR-27a-5p expression was higher in CRPC than in MNPT (morphologically normal prostate tissue) and that miR-27a-5p downregulation was associated with an increased ERGF/Akt1/mTOR oncogenic signaling axis [120]. Interestingly, the results also showed decreased expression of ERGF, phosphorylated Akt1 and mTOR in PC3 cells compared with LNCaP cells [120]. Mechanistically, increased miR-27a-5p directly targets Akt1 and mTOR within the ERGF signaling axis and decreases the expression of ERGF, phosphorylated Akt1 and mTOR [120]. YAP and LATS2 proteins are major downstream effectors of the Hippo pathway, and their abnormal expression has been observed in multiple human cancers, including CRPC [136,137]. Guo et al. reported that the expression of miR302/367 cluster members (miR-302a, miR-302b, miR-302c, miR-302d and miR-267) increased markedly in androgen-insensible PCa cell lines (PC3 and C4-2B) compared with androgen-sensitive cell lines (LNCaP). The underlying mechanism by which the miR-302/367 cluster promotes CRPC development lies in the direct binding of miR-302/367 to the LATS2 3′UTR, a pivotal tumor suppressive effector in the Hippo signaling pathway that exerts a negative effect [138]. In addition, miR-10a could inactivate the Hippo pathway via suppressed transcription of YAP and its target genes, thus playing a tumor-suppressor role in PCa [139].

## 5. Noncoding RNAs and AR-Related Signaling

### 5.1. AR Structure and Functions

AR is an androgen-activated steroid hormone receptor produced by the AR gene, which is located on the long arm of the X chromosome [140,141]. The ligand-dependent nuclear transcription factor controls the expression of a series of genes associated with several cancers, especially prostate cancer and breast cancer [142]. Similar to other nuclear hormone receptors, the full-length AR is mainly composed of four functional motifs: (1) highly conserved LBD (the ligand-binding domain), which possesses the property of specific androgen recognition and binding and moves from an inactive state to a transcriptionally active state once combined as a molecular switch [143]; (2) the best conserved DBD (DNA-binding domain) is mainly responsible for DNA binding and receptor dimerization [144,145]; (3) the NTD (N-terminal transactivation domain), a primary effector region of transcriptional activity achieved by the interaction of NTD at AR with coactivators [146]; (4) the hinge region is a poorly conserved connection between the DBD and LBD harboring a nuclear localization signal (NLS) [147].

The AR signaling pathway is initiated by androgen (testosterone and its active form, dihydrotestosterone) after binding to AR. Then, AR separates from Hsp90 and forms an AR dimer, which elicits its translocation into the nucleus [148]. AR signaling plays a pivotal role in the development and growth of the normal prostate, BPH (benign prostatic hyperplasia) and prostate cancer [2].

**Figure 1:**
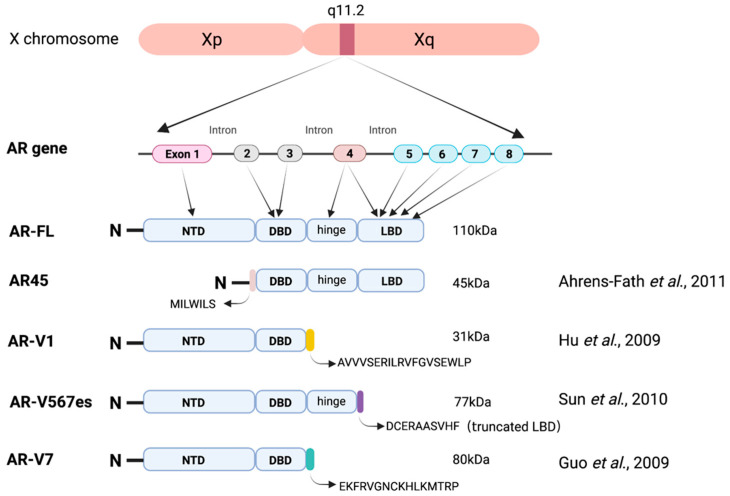
Full-length androgen receptor and its truncated splicing variants. See Refs. [50,54,151,152].

Alternative splicing is a ubiquitous regulatory mechanism that brings about the production of proteome complexity from a limited number of genes [149]. Not surprisingly, at least 20 AR variants (AR-Vs) have been identified thus far (Figure 1) [74,76,150]. For instance, AR45 is a naturally occurring AR variant and has been found in multiple normal tissues, including heart, breast and prostate tissue [151]. Interestingly, studies have shown that AR45 may inhibit the transcriptional activity of full-length AR and biologically reduce androgen-sensitive LNCaP cell growth due to its function as a dominant-negative inhibitor of AR-FL [152]. Another androgen splice variant, AR-V1, could heterodimerize with AR-FL, even in the absence of androgen. This interaction could inhibit the ability of AR-FL to confer castration-resistance cells growth, which suggested that AR-V1 could act as a negative regulator of AR-FL [153]. Yet despite all that, the molecular mechanisms of the interaction between AR-V1 and AR-FL remain unclear. AR-V7, the most extensively studied and the best-characterized AR variant, is highly related to various biological functions, particularly anticancer treatment resistance and poor clinical outcomes [11,154,155]. Structurally, AR-V7 is a truncated isoform lacking the LBD of AR and remains constitutively active even after castration, thus representing an intrinsic mechanism of resistance to ADT [156]. Indeed, increased expression of AR-V7 mRNA and protein can be detected in both primary and metastatic tissues of CRPC patients, in contrast to the undetectable level of AR-V7 in benign prostatic hyperplasia and primary PCa patients with no endocrine therapy, which supports the driving role of AR-V7 in the occurrence and progression of CRPC [155,157]. Similarly, AR-V567es (aka AR-V12) could promote castration-resistance growth of PCa cells in the absence of ligand and is associated with resistance to both abiraterone and enzalutamide [158]. In cells co-expressing AR-V567es and AR-FL, AR-V567es could interact with AR-FL and induce the nuclear translocation of AR-FL in a ligand-independent manner or in an androgen depleted condition [159]. In addition, AR-V567es has also been showed to be capable of regulating canonical androgen-responsive genes in the absence of the AR-FL signaling [160].

### 5.2. AR-Related Signaling in CRPC

**Figure 2:**
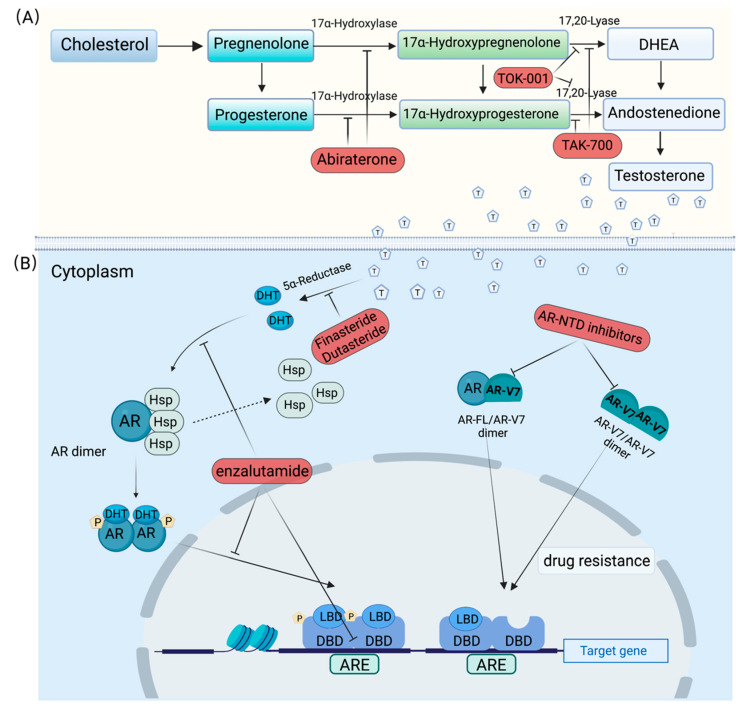
Inhibitors in the treatment of PCa. (**A**) Androgen biosynthesis pathway. All steroid hormone synthesis follows the conversion of cholesterol to pregnenolone, which can subsequently progress down the androgen formation pathway, or be converted to progesterone. CYP17 catalyzes two key reactions involved in the production of sex steroids, which occur sequentially; the 17α-hydroxylase activity typically converts pregnenolone to 17α-hydroxypregnenolone and progesterone to 17α-hydroxyprogesterone, while the 17,20-lyase activity converts 17α-hydroxypregnenolone to DHEA and 17α–hydroxyprogesterone to androstenedione. The DHEA and androstenedione may be subsequently transformed to testosterone by other enzymes. (**B**) Canonical AR signaling pathway and AR-V7 signaling. The inactive AR primarily exists in the cytoplasm and binds to heat shock proteins. Androgen binding to the AR induces conformational changes in the ligand-binding domain and heat-shock protein dissociation from the AR. The transformed AR undergoes dimerization, phosphorylation and translocation to the nucleus. The translocated receptor dimer binds to androgen response elements located in the promoter or enhancer region of AR target genes, leading to the transactivation of AR-regulated gene expression. Abbreviations: DHEA, dehydroepiandrosterone; DHT, dihydrotestosterone; Hsp, heat-shock protein; AR, androgen receptor; LBD, ligand-binding domain; DBD, DNA-binding domain; ARE, androgen receptor element.

Aberrant AR signaling is fundamental to prostate cancer growth, metastasis, metabolic reprogramming and ultimately to its lethality [161,162,163]. Given this, androgen ablation therapy is the first line of defense for patients with prostate cancer and improves survival outcome [164]. However, because these treatments do not effectively block the production of androgen from adrenal, prostate and other tissues, a transition to castration resistance conditions is usually unavoidable. Research over the past few decades has revealed multiple mechanisms involved in CRPC and remains to be elucidated further; however, it is certainly known that the sustained activation of AR signaling program frequently underlies the development of CRPC [165]. Given this, the androgen biosynthesis and androgen signaling pathways have been identified as important targets for the development of anti-androgen drugs (Figure 2) [12,13].

In the test, the last step of androgen biosynthesis involves two critical sequential reactions that are catalyzed by a single enzyme, the cytochrome P450 monooxygenase 17α-hydroxylase/17,20-lyase (collectively referred to as CYP17) [166]. Obviously, inhibition of the critical enzymes that catalyze the biosynthesis of androgen could stop androgen production from all sources, therefore, potentially providing an effective treatment for patients with PCa [167]. As a class of key catalytic enzymes in androgen biosynthesis, CYP17 is currently an important target for PCa treatment, and several CYP17-inhibiting drugs have been developed. Abiraterone, a small molecule derived from the structure of pregnenolone, is the first to be used in clinical practice in several new drugs developed to target adrenal androgen production. It could irreversibly inhibit the catalytic activity of CYP17, which was highly expressed in CRPC, and has been shown to reduce the serum testosterone levels to below a detection threshold of 1 ng/dL [168,169]. Interestingly, abiraterone has been found to inhibit other AR pathway targets, even including the anti-AR activity due to its steroidal structure. However, multiple mechanisms of maintaining AR signaling were observed in the abiraterone-treated PCa, including upregulated expression of AR-FL and ligand-independent AR variants, which leads to abiraterone-resistance in clinical practice and promotes the development of more potent CYP17 inhibitors [170]. TOK-001 and TAK-700, next-generation CYP17 inhibitors, were found to be selective for C17-20 lyase inhibition and exhibited suppressive effects on androgen biosynthesis. In addition, the two inhibitors also cause down-regulation of AR protein expression, which papers attribute to their anti-tumor efficacy.

Enzalutamide is a second-generation competitive inhibitor of AR. It weakens the AR pathway on three levels—through inhibition of androgen binding, nuclear translocation and DNA binding—and thus induces apoptosis, which makes it an attractive option in CRPC [171]. Finasteride is a competitive and specific inhibitor of 5α-reductase, similar in structure to testosterone but has no affinity for AR [172]. Finasteride was originally developed as a pharmacological therapy for BPH (benign prostate hyperplasia) to reduce prostate volume and treat symptoms associated with BPH [173]. Researchers later found that finasteride could be used as a chemopreventive for PCa, for which it was approved for clinical trials and use in 1992 [174,175]. Dutasteride is another inhibitor of 5α-reductase for inhibiting the conversion of testosterone to DHT (dihydrotestosterone). Dutasteride is distinguished from finasteride in that it inhibits both type 1 and type 2 5α-reductase. Therefore, dutasteride is likely to significantly reduce DHT levels when compared with finasteride [176].

Currently, the usual first-line therapies for CRPC are abiraterone and enzalutamide. These AR-targeting agents can extend the survival of patients with CRPC for an extra 5 months but are not curative due to a series of drug resistance mechanisms, including restored androgen receptor signaling, androgen receptor splice variants, androgen receptor bypass signaling and complete androgen receptor independence [177]. Accordingly, previous and recent studies all showed a strong correlation between AR-V7 levels and resistance to enzalutamide and abiraterone [11,178,179]. These findings are in accordance with the special structure of AR-V7, which lacks the LBD, the direct target of enzalutamide and the indirect target of abiraterone. Interestingly, niclosamide, an anti-helminthic drug, was confirmed to re-sensitize enzalutamide-resistant cells to enzalutamide treatment by specifically inducing the degradation of AR-V7 [180,181].

### 5.3. AR-Related ncRNAs in CRPC

**Figure 3:**
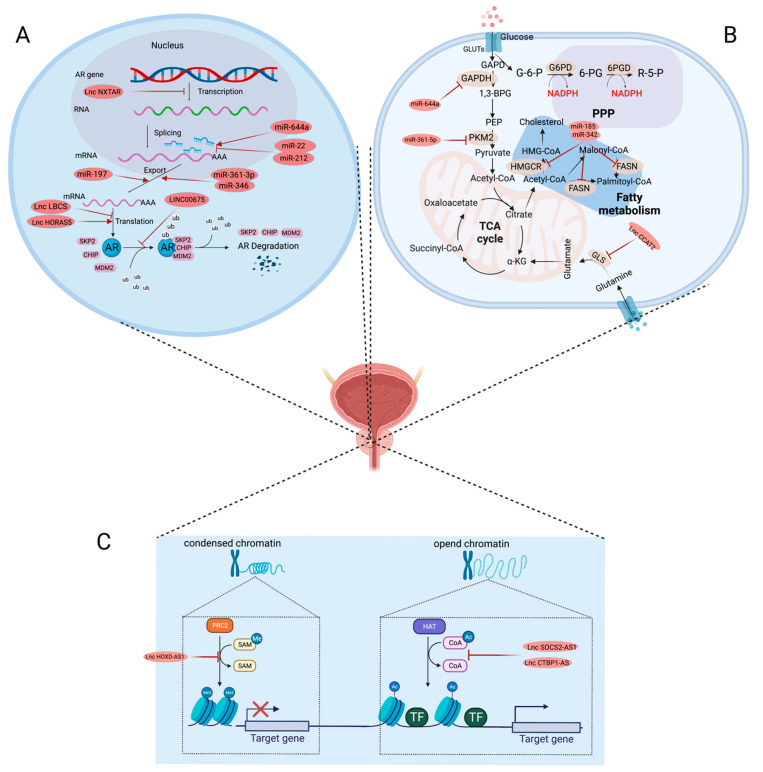
The major contribution of miRNA- and lncRNA-mediated mechanisms, including AR protein regulation, cancer metabolism and epigenetic modification, to the occurrence of CRPC. (**A**) AR protein production and ubiquitin-mediated degradation processes. Key steps were inhibited or promoted by miRNAs, and lncRNAs are shown in red. (**B**) Network of several metabolic pathways involved in glycolysis, glutaminolysis, fatty acid metabolism and the pentose phosphate pathway. Key enzymes in these metabolic pathways were inhibited by miRNAs, and lncRNAs are shown in red. (**C**) Metabolic pathways contributing to histone methylation and acetylation. These two histone epigenetic modifications were inhibited by lncRNAs, as shown in red. Abbreviations: SKP2, S-phase kinase associated protein 2; CHIP, carboxyl terminus of HSC70-interacting protein; GAPDH, glyceraldehyde-3-phosphate dehydrogenase; 1,3-BPG, 1,3-bisphosphoglycerate; PEP, phosphoenolpyruvate; PKM2, pyruvate kinase M2; G-6-P, glucose-6-phosphate; 6-PG, 6-phosphoglukconate; R-5-P, ribose-5-phosphate; G6PD, glucose 6-phosphatedehydrogenase; 6PGD, 6-phosphogluconate dehydrogenase; HMGCR, hydroxymethylglutaryl-CoA reductase; FASN, fatty acid synthase; GLS, glutaminase; PRC2, polycomb-repressive complex 2; SAM, S-adenosyl methionine; HAT, histone acetyltransferase.

Over the past decades, noncoding transcripts have been increasingly recognized as targets or mediators of the AR signaling axis and may play a central role in the enhancement and maintenance of AR signaling activity (Table 2) [37,52,182]. Therefore, the identification of CRPC-associated ncRNAs via bioinformatics analysis or clinical sample testing is important for the development of potential therapeutic targets. Recently, several lncRNAs have been reported to engage in regulating AR expression at the post-transcriptional level (Figure 3). For instance, LINC00675, also known as TMEM238L, has been reported to be dysregulated in several cancers, including gastric cancer, colorectal cancer and cervical cancer [183,184,185]. Recently, a study reported that LINC00675 was more highly enriched in CRPC tissues or cells than in primary PCa tissues or cells, indicating its correlation with CRPC [58]. Functionally, LINC00675 overexpression promoted, whereas knockdown attenuated, cell viability, migration and EMT of LNCaP-SF and LNCaP-C4–2b, two androgen-independent cell lines, showing its relevance to PCa progression [58]. Further experiments found that LINC00675 could stabilize the AR protein by prohibiting AR ubiquitination. This stability function is based on a competitive combination mechanism, whereby LINC00675 could bind to the NTD of AR to damage the interaction between AR and MDM2 (mouse double minute 2), a kind of E3 ubiquitin ligase, thus leading to AR protein stability [58]. Similarly, another study identified and termed an unannotated lncRNA, HORAS, by analyzing PCa PDX models and found that HORAS5 was significantly upregulated in castration-resistant PDXs, considerably expressed only in AR-positive CRPC-derived cell lines and promoted the proliferation and migration potential of CRPC-derived cells by maintaining AR activity in the absence of androgen [59]. Conversely, knockdown of HORAS5 caused a significant decline in the expression of AR and its canonical targets, such as KLK3 and KIAA0101 [55]. Nevertheless, the potential mechanisms were not illuminated and need further research. The maintenance of AR mRNA stability is likely due to a posttranscriptional regulatory mechanism because HORAS5 is predominantly localized in the cytoplasm. Moreover, Gu et al. explored CRPC-relevant lncRNAs via transcriptome microarray and found that lncRNA LBCS could function as an AR translational repressor even under low androgen or AR blockade conditions [186]. LncRNA LBCS was markedly downregulated in CRPC cell lines and tissues in comparison with androgen-dependent cells and correlated with tumor stage, Gleason score and progression [186]. Forced expression of LBCS inhibited prostate cancer viability, whereas LBCS depletion sustained prostate cancer viability by blocking AR signaling in the absence of androgen. Mechanistically, LBCS affects AR protein levels by epigenetic regulation. LBCS could function as a protein scaffold by interacting directly with hnRNPK and guiding it to the 5′-UTR of AR mRNA, thus inhibiting AR translation efficiency, which consequently relieves PCa progression and castration resistance [186]. A novel tumor-suppressor, lncRNA NXTAR, has recently been found to suppress AR expression through recruiting EZH2 methyltransferase. Interestingly, the loss of AR could promote NXTAR expression in turn by allowing GCN5 acetyltransferase to bind to the *NXTAR* upstream region [117]. This negative feed regulation between NXTAR and AR partly explains lower levels of NXTAR in AR-positive CRPC [117].

Additionally, as AR mRNA contains a long 3′-UTR, it is highly probable that the AR signaling pathway or AR expression is affected by a range of miRNAs (Figure 3). Indeed, a recent study revealed that ectopic expression of miR-644a could obviously reduce AR protein and mRNA levels in both androgen-sensitive and androgen-resistant cell lines through a direct interaction with the 3′-UTR of AR mRNA from nucleotides 340 to 358. Most strikingly, a decrease in both full-length and AR-V7 protein levels by miR-644a was also observed in 22RV1 cells, an androgen-independent cell line [96], underpinning the viewpoint that both AR amplification and AR variants were implicated in the development of CRPC. miR-644a also exerts an inhibitory effect on AR transactivation by directly suppressing the expression of AR coregulators, such as SRC-1, SRC-2, SRC-3, CCND1 and CBP (CREB binding protein) mRNA and protein levels [96]. Moreover, miR-22 and miR-212 were downregulated and could modulate the expression levels of hnRNPH1, AR and AR-v7 in C4-2B cells, an AR-expressing CRPC cell line [197]. Additionally, hnRNPH1 was significantly enriched in AR-positive MDA-PCa-2b and C4-2B cells compared to AR-negative PC-3 cells [197]. Interestingly, in accordance with this result, hnRNPH1 silencing induced the inhibition of cell proliferation in MDA-PCa-2b cells but had no significant inhibitory effect in AR-naive PC-3 cells, implying that AR may participate in the modulation of hnRNPH1 expression. Indeed, further research found that miR-22 and miR-212 inhibitors upregulated hnRNPH1 expression, which in turn promoted the expression of AR and its splice variant AR-v7 under hormone-induced and hormone-deprived conditions, which was mediated through the direct binding of hnRNPH1 to AR associated with the recruitment of SAC-3, an AR coactivator [197]. Unlike classical negative regulation of miRNAs to mRNA by base pairing to the 3′UTR of mRNAs, miRNAs also seem to activate mRNA targets in certain situations. Claire E et al. screened a series of AR-targeting miRNAs by transfecting a miRNA inhibitor library into hormone-responsive and hormone-resistant PC cells and identified by luciferase assays that miR-346, miR-361-3p and miR-197 inhibitors significantly repressed AR reporter activity, which resulted from reduced AR protein and mRNA levels [198]. Additionally, miR-346 and miR-361-3p positively regulate AR-v7 levels, a constitutively active variant of AR mRNA lacking the LBD, in 22RV1 and C42 cells [199]. Moreover, miR-346, miR-361-3p and miR-197 inhibitors were discovered to increase apoptosis and suppress proliferation, EMT, migration and invasion after castration [200]. This positive modulation of AR mRNA and protein may be due to the activatory binding of these miRNAs with the 3′UTR of AR and upregulation of AR 3′UTR activity, which is different from the traditional mode of target inhibition by miRNAs. Another possibility is that other effectors may be involved in the above miRNA-mediated regulation of AR expression.

## 6. ncRNAs and Drug Resistance

Although current targeted drugs for PCa have greatly improved the prognosis of men with PCa, patients also inevitably develop resistance to these therapies. While the exact mechanisms of drug resistance have not been fully elucidated, ncRNAs have been found to play a critical role in reversing ADT resistance and improving the sensitivity of targeted drugs with the deepening understanding of ncRNAs recent years [201]. Therefore, correcting the abnormal expression of ncRNAs could be a promising aspect to reverse the drug-resistance of PCa. miR-199a was downregulated in recurrent PCa and paclitaxel-resistance cell lines [202]. Overexpression of miR-199a could increase paclitaxel sensitivity of PCa cells via targeting YES1 expression [202]. Similarly, it has been found that miR-148a was downregulated in hormone-refractory PCa cells and inhibited multiple malignant behaviors [203]. miR-148a could also increase the sensitive to paclitaxel, which was directly mediated by decreasing the expression of MSK1, a direct target of miR-148a [203]. In addition to affecting paclitaxel sensitive, miRNAs also affect docetaxel resistance. The expression of miR-195 was decreased in docetaxel-resistance PCa cells and miR-195 overexpression could promote cells apoptosis and improve the sensitivity of cells to docetaxel via suppressing CLU expression [204]. Similarly, the expression of miR-204 was lower in chemoresistance PCa tissues when compared to that in chemosensitive PCa tissues. Forced expression of miR-204 could markedly promote cells apoptosis and effectively attenuate docetaxel resistance via suppressing ZEB1 expression [205]. Additionally, Lnc CASC2 could also enhance the sensitivity of PCa cells to docetaxel via positively modulating SPRY2 expression and serving as a ceRNA for miR-183 [206].

## 7. Conclusions and Future Prospects

Although most men with prostate cancer benefit from ADT, progression to a castration-resistant state remains inevitable within 2–3 years of initiation of ADT and this poses a major challenge in clinical management. Therefore, a thorough elucidation of the intrinsic mechanisms of CRPC may help to solve this dilemma. An increasing number of ncRNAs have been identified to be involved in epigenetic modifications, tumor metabolism and AR regulation, which are all closely related to the occurrence and progression of CRPC. Clinically, several studies have recognized their roles as promising diagnostic, prognostic and even therapeutic biomarkers, especially miRNAs on account of their relative stability, tiny size and significant control of gene expression. It is conceivable that ncRNAs signatures in circulating cancer cells or macrovesicles secreted by cancer cells could provide personalized treatment for patients with PCa.

However, despite these favorable prospects, circulating ncRNA detection faces technical challenges due to the low abundance of ncRNA in circulation. Therefore, there is an urgent need for other more accurate and convenient non-invasive tests to diagnose PCa. Due to the ease of collection of PCa cells and their direct release into the urethra via the prostate duct, urine is expected to become the non-invasive biomarker test of choice for diagnosis and prognosis. Unfortunately, until now, only Lnc PCA3 has been utilized as a urinary biomarker in the clinical diagnosis of PCa [207]. Additionally, it has recently become obvious that miRNAs interact with their targets in more complex ways than initially realized because an increasing number of researchers have found that many miRNAs engage in both positive and negative feedback loops with their targets. It is also worth pointing out that since the biological functions and mechanisms of action have not been elucidated, drug development targeting ncRNAs must be cautious. For instance, a miRNA might target several genes, even both oncogenes and tumor suppressors, which means that when the expression of one target transcript in malignant tumors increases, the available level of inhibitory miRNA decreases, and this inhibitory effect on other transcripts may be mitigated by target competition. This problem must be given sufficient attention; otherwise, it will place a heavy burden on miRNA-based agent discovery. Further preclinical studies are therefore needed to investigate the interactions between miRNAs and their targets in PCa and the long-term effects of manipulating these subtle networks. Lastly but most significantly, the small molecule inhibitors currently developed to target androgen biosynthesis or AR signaling, as mentioned in Figure 2B, have limitations in delaying the progression of PCa to the castration-resistance stage. Since CRPC is activated by multiple pathways, a single therapeutic strategy is not sufficient to overcome the lethal phenotype of CRPC. However, it is possible for specific ncRNAs to address this restriction; many ncRNAs have been discovered to simultaneously regulate the expression of several members of multiple signaling pathways or cellular processes. Thus, further understanding of the critical regulatory ncRNAs involved in simultaneously targeting multiple pathways, such as the AR signaling pathway and metabolic remodeling, will provide further insight into the development of targeted drugs against highly heterogeneous diseases such as CRPC. In summary, potential ncRNA-directed drugs should undergo extensive preclinical trials to minimize their side effects and toxicity before widespread clinical use.

## Figures and Tables

**Table 1:** The cellular functions and clinical significance of representative ncRNAs in CRPC.

Noncoding RNAs	Expression	Functions	Clinical Significance	Reference
miR-32	Up	Proliferation (+); Apoptosis (−)	Potential marker for aggressive disease	[37,38]
miR-148a	Up	Proliferation (+); Cell cycle (+)	Urine-circulating mir-148a used for detection of PCa	[37]
miR-99a	Down	Proliferation (−)	N/A	[39]
miR-21	Up	Migration (+); Invasion (+); Proliferation (+); Apoptosis (+); Chemo- and radiosensitivity (−)	Associated with biochemical recurrence in low-risk PCa	[40,41,42,43]
miR-221	Up	Proliferation (+); Metastasis (+); EMT (+)	Prognostic marker in high-risk prostate cancer	[44,45]
miR-205	Down	Metastasis (−); Migration (−)	Attenuates progression of prostate cancer	[46,47]
miR-1246	Down	Proliferation (−); Migration (−); Invasion(−); Apoptosis (+)	Correlated with increasing pathologic grade, positive metastasis and poor prognosis	[48]
miR-34a	Down	Metastasis (−)	Potential invasive biomarker; Influences response to docetaxel	[49,50]
LncRNA DRAIC	Down	Migration(−); Invasion (−); EMT (−)	Predicts poor patient outcomes	[51]
LncRNA HOTAIR	Up	Proliferation(+); Invasion (+); Enzalutamide resistance (+)	May be a biomarker of enzalutamide resistance	[52]
LINC00963	Up	Proliferation(+); Apoptosis (−)	May be a novel diagnosis biomarker	[53,54]
LncRNA MALAT-1	Up	Proliferation (+); Migration(+); invasion (+) Arrest in the G0/G1 phases (+)	Correlated with high Gleason score, prostate specific antigen and tumor stage	[55]
LncRNA PCAT29	Down	Proliferation (−); Migration (−); Apoptosis (+)	Associated with higher rates of biochemical recurrence	[56]
LncRNA PCGEM1	Up	Proliferation (−)	Associated with high-risk prostate cancer patients	[57]
LINC00675	Up	Proliferation (+); Migration (+); EMT (+)	Associated with Gleason score	[58]
LncRNA HORAS	Up	Proliferation (+)	Predicts poorer clinical outcomes	[59]
LncRNA PCAT14	Down	Invasion (−)	Associated with Gleason score; predicts disease aggressiveness and recurrence	[60]

**Table 2:** Representative AR-related ncRNAs and molecular mechanisms involved in CRPC.

Noncoding RNAs	Expression	Regulation	Mechanisms	Reference
miR-32	Up	Upregulated by AR	BTG2 is targeted by miR-32	[37]
miR-148a	Up	Upregulated by AR	PIK3IP1 is targeted by miR-148a	[37]
miR-99a	Down	Repressed by AR	EZH2 promotes the repression of miR-99a by AR	[39]
miR-21	Up	Repressed by AR	Drives the downregulation of TGFBR2	[187,188]
miR-221	Up	Abolishes AR-mediated transcription	Mediated by targeting HECTD2	[44]
miR-135	Down	Reduces AR protein and mRNA levels	Interacts with the 3′UTR of AR mRNA	[189]
miR-185	Down	Reduces AR protein and mRNA levels	Interacts with the 3′UTR of AR mRNA; Suppresses BRD8 ISO2 protein	[190]
miR-34a	Down	Reduces AR protein and mRNA levels	N/A	[191]
miR-205	Down	Reduces AR protein and mRNA levels	Interacts with the 3′UTR of AR mRNA	[46]
miR-124	Down	Reduces AR protein and mRNA levels	Interacts with the 3′UTR of AR mRNA	
miR-644a	Down	Reduces both AR-FL and AR-V7 protein levels	Interacts with the 3′UTR of AR mRNA; Suppresses the expression of AR coregulators	[96]
LncRNA DRAIC	Down	Repressed by AR	The occupation of FOXA1 and NKX3-1 at *DRAIC* promoter was abolished	[51]
LncRNA HOTAIR	Up	Upregulates AR protein	Prevents AR ubiquitination and protein degradation by blocking its interaction with MDM2	[52]
LncRNA PCAT29	Down	Repressed by AR	AR binds to the PCAT29 promoter	[56]
LncRNA PCGEM1	Up	Upregulates both AR-FL and AR-V7 proteins; Repressed by AR	*Binds* to the DOT1L-mediated methylated AR N-terminus; Functions as a transcriptional coregulator of the AR	[192,193]
PlncRNA-1	Up	Upregulated by AR; Upregulates AR	Protects AR from miR-34c- and miR-297-mediated suppression	[194]
LncRNA ARLNC1	Up	Upregulated by AR	Directly regulated by AR and modestly regulated by FOXA1; Interacts with 3′UTR of AR mRNA	[195]
LINC00844	Down	Repressed by AR; Enhances AR activity	AR binds to the TSS of LINC00844; Influences AR-regulated transcriptome partly by facilitating the recruitment of AR to the chromatin	[196]
LINC00675	Up	Upregulates AR protein	Stabilizes the AR protein by prohibiting AR from ubiquitination	[58]
LncRNA HORAS	Up	Upregulates AR	Likely due to a posttranscriptional regulatory manner	[59]
LncRNA LBCS	Down	Reduces AR protein	Guilds hnRNPK to the 5′-UTR of AR mRNA and thus inhibits AR translation efficiency	[186]
LncRNA NXTAR	Down	Reduces AR protein	Recruits EZH2 to promoter region of AR mRNA	[117]

## Data Availability

Not applicable.

## Abbreviations

AR: Androgen receptor; ADT: Androgen deprivation therapy; Ac-CoA: Acetyl coenzyme A; AKT: Protein kinase B; ALDOA: Aldolase A; AR-Vs: AR variants; AR-FL: Full-length androgen receptor; BPH: benign prostatic hyperplasia; BRD8 ISO2: Bromodomain containing 8 isoform 2; BTG2: B-cell translocation gene 2; circRNAs: Circular RNAs; CGIs: CpG islands; CRPC: Castration-resistant prostate cancer; ceRNAs: Competing endogenous RNAs; CHUK: Conserved helix-loop-helix ubiquitous kinase; CYP17: Cytochrome P-450 17alpha-hydroxylase/C17,20-lyase; CCND1: Cyclin D1; CBP: CREB binding protein; DNMT1: DNA methyltransferase 1; DNMT3A: DNA methyltransferase 3A; DNMT3B: DNA methyltransferase 3B; DBD: DNA-binding domain; DOT1L: DOT1-like histone lysine methyltransferase; EMT: Epithelial-mesenchymal transition; EZH2: Enhancer of zeste homolog 2; FKBP51: FK506-binding protein 51; FOXA1: Forkhead box A1; GAC: Glutaminase isoform C; GAPDH: Glyceraldehyde-3-phosphate dehydrogenase; GLUT1: Glucose transporter type 1; GLUT3: Glucose transporter type 3; Hsp90: Heat shock protein 90; HECTD2: HECT domain E3 ubiquitin protein ligase 2; HKII: Hexokinase II; hnRNPK: Heterogeneous nuclear ribonucleoprotein K; hnRNPH1: Heterogeneous nuclear ribonucleoprotein H1; LATS2: Large tumor suppressor kinase 2; LBD: Ligand-binding domain; lncRNAs: Long noncoding RNAs; MNPT: Morphologically normal prostate tissue; mTOR: Mechanistic target of rapamycin; mCRPC: Metastatic castration-resistant prostate cancer; mPCa: Metastatic prostate cancer; miRNAs: MicroRNAs; MDM2: Mouse double minute 2; MS: Mass spectrometry; ncRNAs: Noncoding RNAs; NTD: N-terminal transactivation domain; NLS: Nuclear localization signal; NKX3-1: NK3 homeobox 1; PCa: Prostate cancer; PTMs: Posttranslational modifications; PI3K: Phosphatidylinositol 3-kinase; PHLPP: PH domain leucine-rich repeat rotein phosphatase; PIK3IP1: PI3K-interacting protein 1; PGI: Phosphoglucose isomerase; PKM2: Pyruvate kinase M2; SAM: S-adenyl methionine; SRC-1: Steroid receptor coactivator-1; SRC-2: Steroid receptor coactivator-2; SRC-3: Steroid receptor coactivator-3; TCA cycle: Tricarboxylic acid cycle; TGFBR2: Transforming growth factor-beta receptor 2; TSS: Transcription start site; UTR: Untranslated region.
